# MDR1 Inhibition Reverses Doxorubicin-Resistance in Six Doxorubicin-Resistant Canine Prostate and Bladder Cancer Cell Lines

**DOI:** 10.3390/ijms24098136

**Published:** 2023-05-02

**Authors:** Eva-Maria Packeiser, Leoni Engels, Ingo Nolte, Sandra Goericke-Pesch, Hugo Murua Escobar

**Affiliations:** 1 Department of Small Animal Medicine and Surgery, University of Veterinary Medicine Hannover, Foundation, 30559 Hannover, Germany; eva-maria.packeiser@tiho-hannover.de (E.-M.P.);; 2 Unit for Reproductive Medicine—Clinic for Small Animals, University of Veterinary Medicine Hannover, Foundation, 30559 Hannover, Germany; 3 Department of Medicine, Clinic III, Hematology, Oncology and Palliative Medicine, University Medical Center Rostock, 18057 Rostock, Germany

**Keywords:** doxorubicin, chemoresistance, MDR1, ABCB1, cell line, dog, prostate cancer, bladder cancer

## Abstract

Acquired chemoresistance during chemotherapy, often accompanied by cross- and multi-resistance, limits therapeutic outcomes and leads to recurrence. In order to create in vitro model systems to understand acquired doxorubicin-resistance, we generated doxorubicin-resistant sublines of canine prostate adenocarcinoma and urothelial cell carcinoma cell lines. Chemoresistance to doxorubicin, cross-resistance to carboplatin, and the reversibility of the acquired resistance by the specific MDR1-inhibitor tariquidar were quantified in metabolic assays. Resistance mechanisms were characterized by expression of the efflux transporters MDR1 and RALBP1, as well as the molecular target of doxorubicin, TOP2A, with qPCR and Western blotting. Six out of nine cell lines established stable resistance to 2 µM doxorubicin. Drug efflux via massive MDR1 overexpression was identified as common, driving resistance mechanism in all sublines. MDR1 inhibition with tariquidar extensively reduced or reversed the acquired, and also partly the parental resistance. Three cell lines developed additional, non-MDR1-dependent resistance. RALBP1 was upregulated in one resistant subline at the protein level, while TOP2A expression was not altered. Combination therapies aiming to inhibit MDR1 activity can now be screened for synergistic effects using our resistant sublines. Nevertheless, detailed resistance mechanisms and maintained molecular target expression in the resistant sublines are still to be examined.

## 1. Introduction

Canine prostate cancer, regardless of whether adenocarcinoma (PAC) or the frequent urothelial or transitional cell carcinoma (TCC) of the prostate, is often diagnosed at a late stage and with a poor outcome [1]. In particular, PAC is comparable with metastatic, castration-resistant prostate cancer (MCRP) in men [2], as it does not express the androgen receptor and effective treatment options have not yet been elucidated [3,4,5]. Apart from prostate cancer, chemoresistance limits therapeutic outcomes in a majority of tumor entities in both humans and dogs. Primary tumors can either be naturally resistant or acquire specific features under selective pressure during chemotherapy, causing relapse after initially successful cytoreduction. In this process, tumor cells can resort to various mechanisms to overcome cytostatic effects, such as increased efflux and metabolism of the drug, DNA repair, autophagy, apoptosis resistance, stem cell phenotype, and deregulation or mutation of the molecular target itself [6,7,8]. 

Doxorubicin is an effective and thus commonly used chemotherapeutic drug in canine oncology [9]. Intercalation into DNA, disruption of DNA repair, and generation of free radicals as its methods of action make it one of the most potent chemotherapeutic drugs [10]. Specifically, doxorubicin interferes with TOP2A, which uncoils packed DNA during transcription and replication by single strand break and reconnection [11]. 

Efflux transporters are membrane proteins, which play a key role in excretion of potentially harmful substances and participate in the barriers between the bloodstream and the brain or testis. P-glycoprotein, also known as MDR1 and encoded by *ABCB1,* belongs to the ABC family and is a well-known multidrug-resistance protein with a broad spectrum of substrates in both dogs and humans [12]. It is overexpressed in a lot of tumor entities, including canine prostate cancer and in dogs with B-lymphoma relapse after doxorubicin treatment [5,13]. Likewise, doxorubicin-resistant cell lines show high MDR1 levels [14,15,16]. 

Another potential efflux transporter of doxorubicin, not belonging to the ABC family and overexpressed in various human tumor cell lines as well as in lung and colorectal cancer, is RALBP1 [17,18]. As a downstream effector of RALA and RALB [19], it participates in EGFR-endocytosis and cell cycle regulation [20]. Besides its potential role in multi-resistance, RALBP1 is associated with tumorigenesis, tumor cell proliferation, migration, invasion, and radiation resistance [17].

To characterize and develop strategies to overcome acquired doxorubicin resistance, cellular models are powerful in vitro tools [21]. However, while several human tumor cell lines with acquired doxorubicin-resistance are available [22], the number of canine ones is limited [15,16,23]. Specifically, prostate cancer cell lines exist exclusively for the species rat [24]. Thus, the aim of our study was to generate and characterize sublines with acquired doxorubicin resistance from a panel of canine PAC and TCC cell lines. We further identified MDR1 overexpression as a major resistance mechanism and quantified resistances to doxorubicin and carboplatin, proliferation, and invasive behavior. 

## 2. Results

### 2.1. Six out of Nine Cell Lines Became Resistant to 2 µM Doxorubicin

Of the nine PAC and TCC cell lines used, six (Adcarc0846, Adcarc1508, Adcarc1511.1, TCC0840, TCC1509, and TCC1506) achieved a final resistance to 2 µM doxorubicin (Table 1). To these six resistant cell lines, the suffix -doxo was added to distinguish them from the parental cell lines without the suffix. Adcarc0846, Adcarc1508, and TCC1509 were resistant in 28–32 weeks, whereas the other three cell lines took 40, 48, or even 64 weeks to sustain constant proliferation when being exposed to 2 µM doxorubicin. 

### 2.2. Resistance Increased Drastically to Doxorubicin, without Establishing Cross-Resistance to Carboplatin

**Figure 1:**
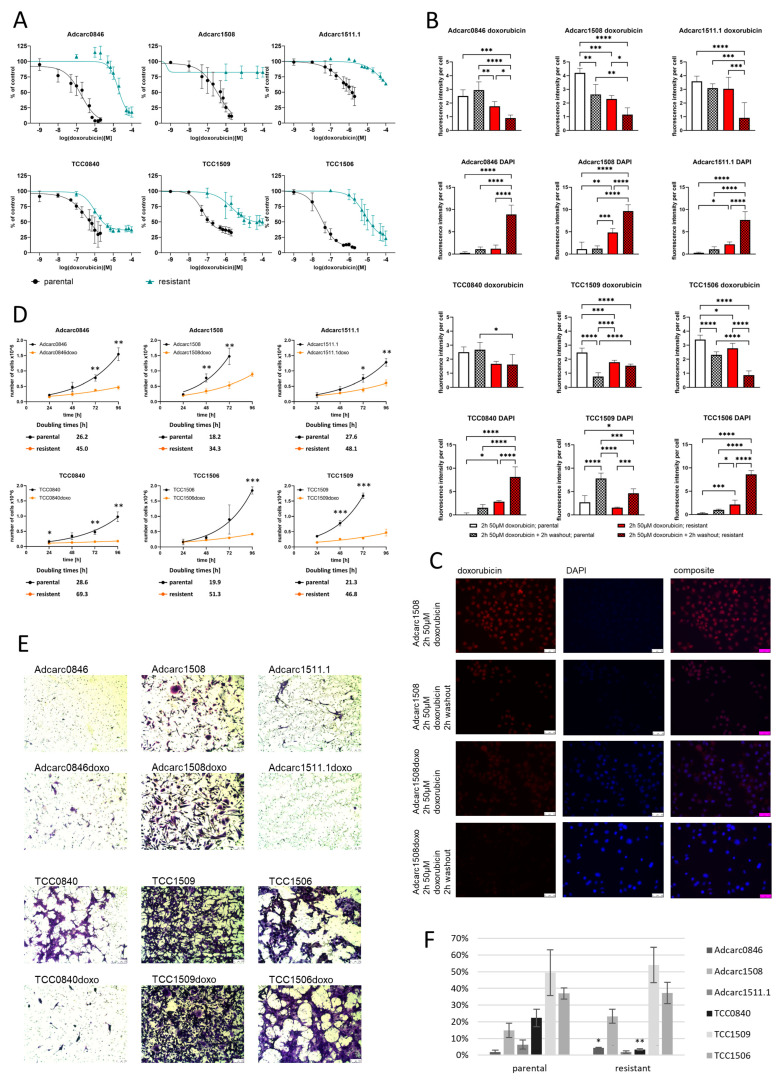
Comparative cell biological characterization of parental and resistant cell lines. (**A**) Metabolic activity of parental and resistant cell lines after exposure to increasing concentrations of doxorubicin for 72 h. Data of the parental cell lines was taken from a previous publication [25]. (**B**) Fluorescence intensities of doxorubicin and DAPI nuclear staining after exposure to 50 µM doxorubicin and after another washout period in doxorubicin-free medium. * *p* ≤ 0.05; ** *p* ≤ 0.01; *** *p* ≤ 0.001; **** *p* ≤ 0.0001. (**C**) Exemplary images of Adcarc1508 and Adcarc1508doxo after exposure to 50 µM doxorubicin and after another washout period in doxorubicin-free medium, 400-fold magnification, scale bar = 50 µM. (**D**) Growth curves and doubling times of parental and resistant cell lines. Asterisks indicate significant differences between parental and resistant sublines; * *p* ≤ 0.05; ** *p* ≤ 0.01; *** *p* ≤ 0.001. (**E**,**F**) Invasive potential of parental and resistant cell lines, based on serum-starved cells, that invaded a second medium compartment containing 10% serum through an artificial basement membrane. Asterisks indicate significant differences between parental and resistant sublines; * *p* ≤ 0.05; ** *p* ≤ 0.01, scale bar = 250 µM.

Compared to the parental cell lines [25], the resistance increased drastically in the generated sublines. In Adcarc1508doxo and Adcarc1511.1doxo, metabolic activity did not decrease below 50%, which is why IC50 values for these cell lines could not be calculated (Figure 1A, Table 1). In general, 20–80% metabolic activity was still recordable at the highest doxorubicin concentration of 100 µM in all resistant cell lines. For Adcarc0846, TCC0840, TCC1509, and TCC1506 –doxo sublines, IC50 values increased by factors of 104, 2, 37, and 227, respectively. Adcarc1508doxo, generated from Adcarc1508 with the second highest resistance within the parental cell lines, also became the most resistant cell line, as its metabolic activity was not reducible below 80%. TCC0840 and TCC0840doxo, on the other hand, were the second most resistant among the parental and most sensitive of the resistant cell lines. As acquired chemoresistance is often accompanied by cross- and multi-resistances, we additionally quantified the susceptibility to carboplatin, another frequently used chemotherapeutic in veterinary medicine. None of the –doxo sublines developed cross resistance towards carboplatin, as IC50 values did not differ by factors > 2 (Table 1). 

### 2.3. The Doxorubicin Resistance Is Based on Drug Efflux

In order to visualize a potential drug efflux, we used doxorubicin’s red autofluorescence and performed fluorescence imaging after incubation with doxorubicin and after an additional washout period. Doxorubicin accumulated in the nuclei of parental cells, as clearly visible by doxorubicin’s red autofluorescence (Figure 1B,C, Supplementary Figure S1). Furthermore, the DNA in most nuclei was saturated by doxorubicin binding, thus DAPI stained only few nuclei. In contrast, less doxorubicin was detected in the resistant cell lines and free DNA binding sites were left for DAPI staining. After a 2 h washout period in doxorubicin-free medium, the situation in the parental cells did not change, whereas the resistant cells eliminated most of the doxorubicin. This effect was significant in all resistant cell lines, except from TCC0840 and TCC1509. Notably, Adcarc1509, TCC1509, and TCC1506 parental cell lines already showed a significant doxorubicin efflux. 

### 2.4. Resistant Sublines Proliferate Slower Than Parental Cell Lines

Another examined parameter, potentially affected by the acquired chemoresistance, was pace of proliferation. Generated resistant sublines proliferated significantly slower than their parental equivalents, leading to longer times needed for population doubling (Figure 1D). With 34 h, Adcarc1508doxo had the shortest doubling time amongst the resistant cell lines, compared to TCC0840 with the longest doubling time of 69 h. 

### 2.5. TCC1509doxo and TCC1506doxo Remained Highly Invasive, while TCC0840doxo Lost Its Invasive Potential

The resistant sublines of TCC1509 and TCC1506 had a similarly high invasive potential compared with their parental cell lines [26] (Figure 1E,F), while TCC0840doxo lost the ability to invade through an artificial basement membrane. The area covered by invading cells stayed below 10% for Adcarc0846 and Adcarc1511.1, while Adcarc1508doxo became more invasive in trend. 

### 2.6. At the mRNA Level, ABCB1 Was Upregulated and Highly Expressed in All Resistant Cell Lines

**Figure 2:**
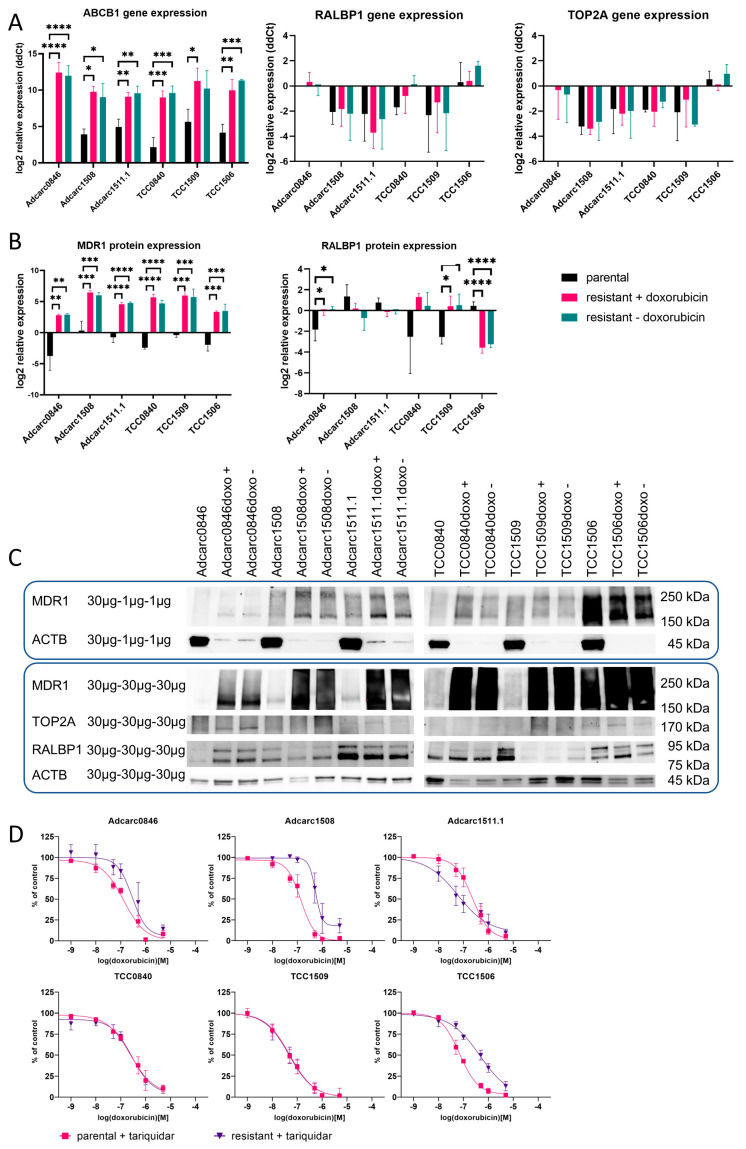
Comparative molecular biological characterization of parental and resistant cell lines. (**A**) *ABCB1*, *RALBP1*, and *TOP2A* gene expression in parental and resistant cell lines, cultured in the presence (+) or absence (−) of 2 µM doxorubicin. * *p* ≤ 0.05; ** *p* ≤ 0.01; *** *p* ≤ 0.001; **** *p* ≤ 0.0001. (**B**) Quantitative analysis of MDR1 and RALBP1 protein expression by Western blot. Asterisks indicate significant differences between parental and resistant sublines; * *p* ≤ 0.05; ** *p* ≤ 0.01; *** *p* ≤ 0.001; **** *p* ≤ 0.0001. (**C**) Western blot images of MDR1, TOP2A, and RALBP1; for ABCB1 quantification, the protein load per lane of the resistant cell lines was reduced to 1 µg, due to the extreme upregulation. (**D**) Metabolic activity of parental and resistant cell lines after exposure to increasing concentrations of doxorubicin for 72 h in the presence of the MDR1 inhibitor tariquidar.

For a molecular biological characterization of the established doxorubicin resistance, the expression levels of efflux transporters MDR1 (encoded by *ABCB1*) and RALBP1, as well as the enzyme TOP2A as molecular target of doxorubicin, were measured at the mRNA and protein level. Relative *ABCB1* gene expression among the parental cell lines was lowest in Adcarc0846 (Figure 2A), which was significant in all cell lines but TCC0840. TCC1509 showed the highest *ABCB1* gene expression of all parental cell lines, significantly higher compared to Adcarc0846 and TCC0840. When comparing parental and resistant cell lines, cultured either with or without doxorubicin, *ABCB1* gene expression was upregulated by a log2fold change between 4.0 in Adcarc1511.1 (*p* < 0.01) and 12.7 in Adcarc0846 (*p* < 0.0001). This was significant in all comparisons except from TCC1509 vs. TCC1509doxo cultured without doxorubicin. Culturing the resistant cell lines without doxorubicin for three days did not reduce the *ABCB1* gene expression. In contrast to *ABCB1*, gene expression of *RALBP1* and *TOP2A* was not altered in the resistant cell lines compared with the parental equivalents, neither in presence, nor in absence of doxorubicin.

### 2.7. At the Protein Level, ABCB1 Was Upregulated and Highly Expressed in All Resistant Cell Lines

ABCB1 protein expression among the parental cell lines was lowest in Adcarc0846 and highest in Adcarc1508 (Figure 2B,C). All resistant cell lines showed upregulated ABCB1, with highest significances (*p* < 0.0001) in Adcarc1511.1 and TCC0840. The highest ABCB1 protein expression was observed in Adcarc1508doxo and TCC1509doxo, while ABCB1 protein abundance among the resistant cell lines was lowest in Adcarc0846 and TCC0840. RALBP1 was downregulated in TCC1506doxo (*p* < 0.0001) and upregulated in Adcarc0846doxo as well as TCC1509 (*p* < 0.05). TOP2A was not expressed in sufficient abundance for quantification; however, no obvious upregulation was observed. 

As in gene expression, doxorubicin deprivation did not influence protein expression. 

### 2.8. MDR1 Inhibition Reversed the Doxorubicin Resistance in Varying Degrees

As an upregulation of MDR1 expression was detected at mRNA and protein level (Figure 2), we investigated if MDR1 inhibition reverses the acquired resistance by the metabolic MTS assay. The inhibition of the MDR1 transporter by tariquidar influenced the doxorubicin resistance in the different pairs of parental and resistant cell lines individually (Figure 2D, Table 2). 

The IC50 values of parental and resistant TCC0840 under tariquidar exposure were below that of the parental TCC0840 and at a similar level, the same in TCC1509. In Adcarc0846doxo, Adcarc1508doxo, and TCC1506doxo, the IC50 decreased under tariquidar exposure; however, not to the baseline level of the parental equivalents. Tariquidar had no influence on the resistance of parental Adcarc0846 and TCC1506, while the IC50 of parental Adcarc1508 could be further decreased. In the parental and resistant pair of Adcarc1511.1, MDR1 inhibition reduced the chemoresistance of both; however, Adcarc1511.1doxo was even more sensitive than Adcarc1511.1. Additionally, tariquidar reduced the leftover metabolic activity at the highest doxorubicin concentration to approximately 10 to 20% in TCC1506doxo and Adcarc1508doxo, and to approximately zero in all other parental and resistant cell lines. 

## 3. Discussion

Natural or acquired chemoresistance limits the outcome of chemotherapy, leading to treatment failure or remission. A better understanding of resistance mechanisms and cross-resistances, as well as the discovery of prognostic biomarkers, help us to choose an optimal treatment protocol and, therefore, avoid ineffective treatment attempts. Chemo-resistant sublines are excellent tools to examine acquired resistance. With our study, we present six new pairs of parental and doxorubicin-resistant cell lines derived from canine prostate and bladder cancer.

Our cell lines’ chemosensitivities are in the same range with CMT-Stylo, a canine mammary cancer parental cell line, and CMT-Star, its doxorubicin-resistant subline [15], as well as with two resistant human breast cancer sublines [27]. Another concordance with these studies is a large percentage of residual metabolic activity at the highest doxorubicin concentrations, which indicates a population of highly resistant clones. Interestingly, canine lymphoma cell line GL-1 and its subline GL-40 are much more sensitive, and GL-1 lacks a highly resistant subpopulation [16]. Clinical outcomes confirm these observations, as doxorubicin is part of the standard CHOP treatment protocol and the most efficient single-agent chemotherapy for canine lymphoma [28], while the outcome of doxorubicin in mammary cancer and TCC is not satisfactory [29,30]. However, a highly resistant subpopulation in the resistant GL-40 confirms that acquired resistance and recurrence are obstacles to overcome in canine lymphoma as well. 

As observed in fluorescence microscopy, doxorubicin was quickly eliminated by the –doxo sublines. Thereby, drug efflux is more likely than metabolic turnover to the less toxic doxorubicinol, as the latter fluoresces at the same wavelength as doxorubicin [31]. The efflux transporter MDR1 contributed to a large percentage of the acquired resistance in our cell lines, as the transporter was massively upregulated at the mRNA and protein level and its inhibition by tariquidar drastically increased the cytotoxic effect of doxorubicin. Notably, while IC50 values in all resistant and even some of the parental cell lines were extremely high, the combination with tariquidar lowered effective doxorubicin concentrations to therapeutically achievable plasma concentrations [32]. 

As TCC0840doxo and TCC1509doxo behaved according to the parental equivalents when being exposed to tariquidar and doxorubicin, their acquired resistance could be attributed completely to MDR1 upregulation. In contrast, Adcarc0846doxo, Adcarc1508doxo, and TCC1506doxo featured additional, non-MDR1-dependent resistance mechanisms, as their IC50 values were not reduced to a level comparable with the parental cell lines. It remains to be clarified if these sublines consist of distinct clones with separate mechanisms [33], or one multi-resistant clone. Interestingly, Adcarc1511.1doxo was more sensitive than the parental equivalent when exposed to tariquidar. In reverse, a heterogeneous population of tumor cells [34], rich in different resistance mechanisms, allows a high resistance of parental Adcarc1511.1, while clones with high MDR1 expression were selected by our protocol. Additionally, MDR1 upregulation was essential for highly resistant subpopulations in both resistant and parental lines, as they were substantially reduced or even eliminated by doxorubicin combined with tariquidar. Regarding the parental cell lines, Adcarc1508, Adcarc1511.1, TCC0840, and TCC1509 responded to the addition of tariquidar, while Adcarc0846 and TCC1506 did not. Correspondingly, a low baseline ABCB1-expression was confirmed by qPCR, Western blot, and also RNA-Seq data [26] in these two cell lines, which additionally lack a highly resistant subpopulation.

Based on our findings, combination therapies with MDR1 inhibitors could consequently be a promising therapeutic approach. However, despite tariquidar’s reliable MDR1 inhibition in vitro [35], there is just one clinical study with published results at clinicaltrials.gov. Unfortunately, although well tolerated, the combination of tariquidar with docetaxel was not as promising as hypothesized [36]. Similarly, combinations of MDR1 inhibitors and doxorubicin have been tested in two clinical trials in dogs; however, the results are not yet sufficient for final conclusions [8]. Tyrosine kinases, of which solely masitinib and toceranib are licensed for veterinary use in Europe, are other substances discussed as potential MDR1 substrates and inhibitors. In canine lymphoma cells, masitinib reduced doxorubicin resistance, although the effect of masitinib itself on the used cell line was only minor [37]. Particularly interesting for clinical trials are synergistic effects, as shown for the combination of crizotinib and doxorubicin on human hepatocellular carcinoma cells [38]. The most promising candidates for consecutive in vitro studies in dogs are cell lines expressing the specific molecular targets of masitinib. Of these, our parental cell lines lack the major target c-Kit; however, masitinib proved to have effects on the expressed targets LYN, PDGFRA, and PDGFRB [39]. Whether the resistant sublines maintained the tyrosine kinase receptor expression [26] remains to be investigated. Nevertheless, special care has to be taken in dogs with MDR1 mutation. Homozygote dogs completely lack MDR1 function, which makes them highly susceptive to adverse effects by MDR1 substrate drugs [12]. 

Although MDR1 overexpression appears to be the driving mechanism for doxorubicin resistance in our study, as well as in others [14,23,40], it is not exclusive. Tegze et al. generated 16 doxorubicin-resistant sublines from two human mammary cancer cell lines [27]. Notably, not all overexpress *ABCB1* or show increased MDR1 activity. As expected, since platinum-based agents are not substrates of MDR1 [41], ours as well as sublines from Tegze et al. with high MDR1 activity were not cross-resistant. On the other hand, two doxorubicin-resistant sublines with non-MDR1-dependent mechanisms are more resistant to platinum-based agents [27]. It is, therefore, interesting that *ABCB1*-overexpressing, doxorubicin-resistant canine mammary cancer [15] and human osteosarcoma cells [40] relied on multiple resistance mechanisms, as they also showed cross-resistance to platinum-based agents. This again underlines the need for cellular models to better understand multi-resistance.

In our resistant sublines, additional to MDR1, RALBP1 was upregulated in Adcarc0846doxo and TCC1509doxo at the protein level. In a next step, functional analyses are necessary to verify the role of RALBP1 in these two sublines [42]. Contrary to this, TOP2A expression was not related to doxorubicin resistance in our study. Possible further resistance mechanisms might be overexpression of other ABC transporters, such as MRP1 (*ABCC1*) or BRCP (*ABCG2*) [43], mutations in ABC transporter genes [44,45] or *TOP2A* [46], activation of detoxifying enzymes, such as glutathione-S-transferase π (GSTP) [47,48], epithelial to mesenchymal transition [48], a stem cell phenotype [49], apoptosis-resistance [50], and many more. Besides the aforementioned MDR1 overexpression, MicroArray analysis of canine parental CMT-Stylo and doxorubicin-resistant CMT-Star revealed deregulated biological and cellular processes associated with exosomes, translation, cell cycle, and apoptosis. Similar analyses, as already performed for human osteosarcoma cells [40], or comparative RNA sequencing, can provide detailed insights into the acquired resistance mechanisms of our established sublines. 

Besides MDR1 upregulation, a slower proliferation rate was beneficial for surviving high doxorubicin concentrations, a phenomenon which was also observed in canine mammary cancer cells [15]. Possible explanations might be the energetically high effort of MDR1 activity, impaired TOP2A function, or DNA repair due to the DNA-intercalation of doxorubicin. As observed in doxorubicin-resistant MCF-7, epithelial to mesenchymal transition is attributed to acquired chemoresistance [48], which itself is associated with a higher cell mobility and invasiveness [51]. Surprisingly, TCC0840doxo was less invasive than its parental cell line, a finding that is supported by Sahabi et al. in canine mammary cancer cells CMT-Star. Clonal selection of less invasive TCC0840doxo cells might again serve as an explanation. 

Another factor in the acquisition of chemoresistance is the required time. With 20 weeks, CMT-Star is the fastest [15], followed by our cell lines Adcarc1508doxo, Adcarc0846doxo, and TCC1509doxo with 28 to 32 weeks. Thereby, baseline MDR1 expression did not predict the progress. Assuming that these parental cell lines adapt to other chemotherapeutics in a similarly short time, they might be interesting models to study highly adaptive tumors with short times to recurrence. 

One special case is TCC0840, which started with a relatively high resistance and resulted in being the most sensitive –doxo subline. An explanation lies in the case itself, the only non-chemonaïve patient [25]. This ten-year old male neutered Pitbull Terrier was initially treated for B-cell lymphoma. After full completion of the CHOP protocol, including four rounds of doxorubicin, and complete remission, he developed hemangiosarcoma, a mass in the lung, and prostate carcinoma. It therefore appears likely that tumorigenesis in this patient was facilitated by immunosuppression and/or the oncogenic potential of chemotherapy [52,53]. Nonetheless, these tumors were probably primed with a certain spectrum of resistance mechanisms. Although platinum compounds were not included in the CHOP protocol, these mechanisms might explain TCC0840’s high resistance to carboplatin and make it an interesting model for multi-resistance. 

Although the MDR1 overexpression was stable after several days without doxorubicin and freshly thawed stocks could be exposed to 2 µM doxorubicin without problems, we did not examine long-term stability of the acquired resistance. Additionally, an influence of doxorubicin on formazan production in the conducted MTS assay cannot be excluded. Despite their initially high IC50 values, Adcarc1258 and the two PAC metastasis-derived cell lines could not establish resistance to doxorubicin. Interestingly, at resistance concentrations between 64 and 256 nM, MCF-7 overexpressed MRP1 and switched strategy to overexpression of MDR1, GSTP, and a mesenchymal phenotype at 1 µM [48]. It remains to be investigated whether this switch was also critical for Adcarc1258 and the two metastasis cell lines. As McDermott et al. stated, the generation of chemo-resistant cell lines is not always easy, although non-successful attempts are unfortunately not reported [21]. Reasonably, they suggest a pulse-based protocol for intra venously applied drugs, which more closely mimics the situation in vivo. On the other hand, as our protocol was successful for six out of nine cell lines, the other three might as well be of another value. We veterinary oncologists prefer the kind of tumors that do not end in remission.

## 4. Materials and Methods

### 4.1. Generation of Doxorubicin-Resistant Cells

Nine cell lines derived from four prostate adenocarcinoma (PAC), two metastasis of a PAC, and three urothelial carcinomas (TCC) [25] originating from prostate or bladder were cultured in Medium 199 (Life Technologies GmbH, Darmstadt, Germany) containing 10% fetal calf serum (FBS Superior, Biochrom GmbH, Berlin, Germany), 200 IU/mL penicillin, and 200 mg/mL streptomycin (Biochrom GmbH) in humidified air at 37 °C and 5% CO_2_. Over 1.5 years, resistant subpopulations were generated by adding increasing concentrations of doxorubicin to the medium, until final resistance towards the peak therapeutic plasma concentration of 2 µM in dogs [54] was achieved. Doxorubicin (Doxo-Cell 2 mg/mL, Stadapharm GmbH, Bad Vilbel, Germany) was diluted in cell culture medium and stored in aliquots at −20 °C. Stably proliferating cells were passaged at least twice, until the dose was incremented in steps of IC5, IC10, IC15, IC30, IC50, IC70 and, if not already achieved, 0.1 µM, 0.2 µM, 0.5 µM, 1 µM, and 2 µM. Cells sustaining 2 µM doxorubicin were considered resistant and the suffix “doxo” was added to the cell line’s name. 

### 4.2. Visualization of Doxorubicin Efflux by Fluorescence Microscopy

Doxorubicin’s red autofluorescence was used to visualize uptake and efflux by fluorescence microscopy. Therefore, parental and resistant cell lines were seeded at a density of 7500 cells per well in 96 well plates. The next day, they were exposed to 50 µM doxorubicin for 2 h, before being fixed for 20 min in 4% paraformaldehyde, permeabilized in 0.2% Triton X-100 (Sigma-Aldrich Chemie GmbH), washing in PBS, and staining with 4,6-diamidino-2-phenylindole diluted 1:1000 (DAPI, Sigma-Aldrich Chemie GmbH, Munich, Germany). In another approach, in order to visualize a possible doxorubicin efflux, cells were granted a 2-h washout period in regular cell culture medium after the 2 h doxorubicin exposure, before being stained. Fluorescence images were taken at 400-fold magnification utilizing a DMI600 B microscope (Leica Microsystems, Wetzlar, Germany) with LAS AF 2.6.0 software. Fluorescence intensities in the red (doxorubicin) and blue (DAPI) channel were calculated, after background subtraction, using ImageJ 2.0.0.

### 4.3. Quantification of Chemoresistance and Cross-Resistance to Carboplatin

In order to quantify the extent of resistance of the –doxo sublines and compare it with the respective parental ones, half maximal inhibitory concentrations (IC50) on metabolic activity were determined. Therefore, 7500 cells per well were seeded in 96 well plates and exposed to a dilution series of doxorubicin in quadruplicates the next day. For detecting possible cross-resistances towards carboplatin, increasing concentrations of Carbo-Cell 10 mg/mL (Stadapharm GmbH) were applied. Controls were supplied with fresh cell culture medium without solvent control, as both agents were dissolved in sterile water. Concentrations were chosen as follows: doxorubicin 1 nM–100 µM; carboplatin 1 µM–1 mM. After 72 h incubation, the CellTiter 96^®^ Aqueous One Solution Cell Proliferation Assay (Promega GmbH, Mannheim, Germany) was conducted in accordance with the manufacturer’s protocol. A Synergy2 plate reader (BioTek, Bad Friedrichshall, Germany) served to record absorbance at 490 nm. Blanks were subtracted and values were normalized to the respective medium control. IC50 values were calculated with Prism software 8.3.0 (GraphPad Software Inc., San Diego, CA, USA). Results were compared with the previously assessed chemosensitivities of the parental cell lines [25]. Cross resistance to carboplatin was assumed if the IC50 values differed by a factor > 2. 

### 4.4. Chemoresistance under MDR1 Inhibition

The non-competitive MDR1 inhibitor tariquidar was purchased from Selleck Chemicals (Houston, TX, USA) to verify whether the doxorubicin-efflux was MDR1-dependent. Parental and resistant cell lines were seeded in 96 well plates at a density of 7500 cells per well. The next day, medium was replaced by 100 µL medium containing 2 µM tariquidar. After one hour, 100 µL medium with doxorubicin was added, resulting in quadruplicates with a final concentration of 1 µM tariquidar and 1, 10, 50, 100, 500, 1000, 5000, and 10,000 nM doxorubicin. Controls were exposed to DMSO or tariquidar only. After 72 h, the CellTiter 96^®^ Aqueous One Solution Cell Proliferation Assay (Promega GmbH) was carried out. Blanks were subtracted and absorbance values were normalized to the tariquidar-only controls and IC50 values were calculated using Prism software 9.0 (Graphpad Software Inc., La Jolla, CA, USA). 

### 4.5. Calculation of Population Doubling Times

Growth kinetics were assessed as previously described [25]. Cells were seeded in 6 well plates at a density of 200,000 cells per well in presence of 2 µM doxorubicin. After 24, 48, and 96 h, cells from three wells were detached and counted with an automated cell counter (Cellometer Auto-T4, Nexcelom Bioscience LLC, Lawrence, MA, USA). Exponential equations were generated using the formula n(t) = N_0_*r^t^ (n_0_ = cell count after 24 h, r = respective growth rate of each cell line). Finally, doubling times (DT) were calculated as follows: DT[h] = ln(2)/ln(r).

### 4.6. Estimation of Invasive Potential

As described for the parental cell lines [26], cells in serum-free medium were seeded in cell culture inserts with 8 µm pores (Falcon^®^, Corning inc., Corning, NY, USA), coated with basement membrane (Cultrex^®^, Bio-Techne Corp., Minneapolis, MN, USA). The next day, the lower chambers were filled in duplicate with medium containing 10% serum as attractant. Serum-free medium served as negative control. Following 48 h incubation time, non-invasive cells were gently removed from the upper chamber by using moistened cotton swaps. The remaining invasive cells attached to the membranes were fixed in 10% formalin for 10 min, permeabilized in methanol for 20 min, and washed twice in PBS. Finally, the membranes with attached cells were stained for 2 min with 1% crystal violet, washed again three times in PBS, and allowed to dry. Three representative regions of each membrane were photographed with a DMI600 B microscope (Leica Microsystems, Wetzlar, Germany) at 100-fold magnification using the LAS AF 2.6.0 software. The proportion of area covered by invasive cells was determined with ImageJ 1.53c. [55]. 

### 4.7. Expression of Resistance-Associated Genes

In three independent experiments, cells were cultured for 96 h in presence of 2 µM doxorubicin (doxo lines only) or without doxorubicin (all cell lines). Afterwards, cells were washed with ice-cold PBS and harvested using cell scratchers. RNA was isolated with the SV Total RNA Isolation System (Promega GmbH) and quantified photometrically using a NanoPhotometer^®^ NP80 (Implen GmbH, München, Germany). For cDNA synthesis, applying the QuantiTect Reverse Transcription Kit (Qiagen GmbH, Hilden, Germany), 2 µg RNA per 10 µL reaction was used. Primer pairs (Table 3) were designed with the Primer-BLAST (https://www.ncbi.nlm.nih.gov, accessed on 14 July 2022) and purchased from Microsynth AG (Balgach, Switzerland). Specific primer binding was confirmed by sequencing the amplicons (Eurofins Genomics Germany GmbH, Ebersberg, Germany). Reactions were run in triplicates on a LightCycler^®^96 real-time PCR System (Roche Diagnostics GmbH, Penzberg, Germany). Cycling conditions were as follows: 95 °C for 10 min, 45 cycles (95 °C for 10 s, annealing for 10 s, and 72 °C for 10 s), followed by melting. Data analysis was carried out using Roche LightCycler^®^96 software (1.1.0.1320). A two-fold dilution series of pooled cDNA served for calculating primer efficiencies. *GAPDH* and *ACTB* gene expression was more stable compared to *HPRT*, which is why they were chosen as normalization genes. Gene expression values relative to Adcarc0846 were calculated according to an efficiency-corrected model, taking into account both normalization genes [56]. 

### 4.8. Expression of Resistance-Associated Proteins

Cells were incubated as described for gene expression, washed with ice-cold PBS, and harvested in RIPA-Buffer with cOmplete™ protease inhibitor cocktail (Roche Diagnostics GmbH) by scraping. Protein concentrations were assessed by Pierce™ BCA Protein Assay Kit (Thermo Fisher Scientific Inc., Waltham, MA, USA). Without previous cooking, as required by the ABCB1-antibody, proteins (30 µg per lane) were separated by SDS-PAGE (Mini-Protean^®^TGX™ Gels, Bio-Rad Laboratories Inc., Hercules, CA, USA). After blotting onto a nitrocellulose membrane (Trans-Blot^®^ Turbo™ Transfer Pack, Bio-Rad Laboratories Inc.), unspecific binding was blocked by EveryBlot blocking buffer (Bio-Rad Laboratories Inc.). Primary antibodies (Table 4) were incubated over night at 4 °C, followed by secondary antibodies (Table 4) for 1 h at room temperature. Chemiluminescent signals were visualized on a ChemiDoc™ Imager (Bio-Rad Laboratories Inc.). Protein expression was quantified utilizing ImageJ 1.53c [55]. Since ABCB1 signals in the resistant cell lines were overexposed, Western blots were repeated, and the protein amount of the resistant samples was reduced by a factor of 30 to 1 µg per lane. 

### 4.9. Statistics

Experiments, except from fluorescence microscopy, were repeated three times independently. All calculations were conducted with Prism software 9.0 (Graphpad Software Inc.). For qPCR and Western blot analysis, Log2 transformed relative expression values were used. To compare fluorescence intensities as well as gene and protein expression of the parental cell lines with the resistant clones, cultured either with or without doxorubicin, a one-way ANOVA was performed followed by Tukey’s post-hoc test in case of normal distribution (Shapiro–Wilk test). If the data were not normally distributed, the Kruskal–Wallis test was used, followed by Dunn’s multiple comparisons test. 

Regarding cell numbers for doubling times and area covered by cells for invasion assay, parental and resistant cell lines were compared with Student’s t-test because of normally distributed data. 

## 5. Conclusions

Acquired chemoresistance limits the therapeutic outcome in veterinary and human oncology, yet no satisfying treatment strategy for canine prostate cancer exists. In order to create in vitro models to overcome acquired chemoresistance, we generated six doxorubicin-resistant sublines from nine well characterized canine prostate cancer cell lines. Drug efflux by massive MDR1 overexpression was identified as a common driving resistance mechanism in all sublines. Moreover, Adcarc0846doxo, Adcarc1508doxo, and TCC1506doxo developed additional, non-MDR1 dependent resistance mechanisms, which still need to be characterized. MDR1 inhibitors, such as the herein efficiently used tariquidar, reduce or reverse the acquired doxorubicin-resistance. Furthermore, tyrosine kinase inhibitors show potential in reversing MDR1-dependent drug-resistance, while exhibiting antiproliferative effects themselves. Potential synergistic effects can now be investigated with our six pairs of parental and resistant cell lines. However, in advance, the expression signature of molecular targets initially observed in the parental cell lines remains to be confirmed. 

## Figures and Tables

**Table 1:** Used cell lines, halfmaximal inhibitory concentrations (IC50) of doxorubicin and carboplatin and needed time span for the generation of resistant sublines to 2 µM doxorubicin.

Cell Line ^1^	Histological Classification	Time to Resistance	IC50 Parental Doxorubicin ^3^	IC50 Resistant Doxorubicin	IC50 Parental Carboplatin ^3^	IC50 Resistant Carboplatin
TihoDPro**Adcarc1258**	PAC of the prostate	N/A ^2^	0.35 µM	N/A ^2^	97.7 µM	N/A ^2^
TihoDPro**Adcarc0846**	PAC of the prostate	30 weeks	0.18 µM	18.40 µM	106.0 µM	115.3 µM
TihoDPro**Adcarc1508**	PAC of the prostate	28 weeks	0.35 µM	X ^4^	67.7 µM	87.9 µM
TihoDPro**Adcarc1511.1**	PAC of the prostate	48 weeks	1.31 µM	X ^4^	86.1 µM	94.6 µM
TihoDPro**Metadcarc1511.2**	PAC metastasis	N/A ^2^	>2 µM	N/A ^2^	38.3 µM	N/A ^2^
TihoDPro**Metadcarc1511.3**	PAC metastasis	N/A ^2^	>2 µM	N/A ^2^	46.1 µM	N/A ^2^
TihoDProCarc/**TCC0840**	TCC of the prostate	64 weeks	0.49 µM	1.03 µM	129.3 µM	70.1 µM
TihoDPro**TCC1509**	TCC of the prostate	32 weeks	0.06 µM	2.10 µM	33.6 µM	30.7 µM
TihoDUrt**TCC1506**	TCC of the urinary bladder	40 weeks	0.03 µM	7.26 µM	39.8 µM	78.6 µM

^1^ Name parts written in bold are further on used as abbreviated names; ^2^ No resistant subline could be generated; ^3^ Data of the parental cell lines was taken from a previous publication [25]; ^4^ Metabolic activity did not decrease below 50%, so IC50 values could not be calculated.

**Table 2:** IC50 values of parental and resistant cell lines exposed to doxorubicin in the presence of the MDR1 inhibitor tariquidar and IC50 values to carboplatin as potential cross-resistance.

Cell Line	IC50 Doxorubicin with Tariquidar
Adcarc0846	0.13 µM
Adcarc0846doxo	0.29 µM
Adcarc1508	0.14 µM
Adcarc1508doxo	0.54 µM
Adcarc1511.1	0.23 µM
Adcarc1511.1doxo	0.06 µM
TCC0840	0.24 µM
TCC0840doxo	0.28 µM
TCC1509	0.05 µM
TCC1509doxo	0.05 µM
TCC1506	0.07 µM
TCC1506doxo	0.46 µM

**Table 3:** Primer Pairs.

Gene	Sequence (5′-3′)	Amplicon Length	Efficiency	Temperature	Accession Number
*ABCB1*	for GACTCGGGAGCAGAAGTTTGA rev ACCCCGAAGATGTGTGCTTT	90 bp	1.97	57 °C	NM_001003215.2
*RALBP1*	for TGGCATGAAGTGTGAAGGCA rev TCCTCTCTGTCATAGGCTGCT	84 bp	1.94	57 °C	XM_038672657.1
*TOP2A*	for TCAGCCCTTTGGCTCGGTTA rev TTGCAGGACCACCCAGTACC	160 bp	1.93	60 °C	XM_038676554.1 XM_038676553.1
*GAPDH* [57]	for GGCCAAGAGGGTCATCATCTC rev GGGGCCGTCCACGGTCTTCT	228 bp	1.99	60 °C	NM_001003142
*ACTB* [57]	for GCTGTGCTGTCCCTGTATG rev GCGTACCCCTCATAGATGG	98 bp	1.98	60 °C	NM_001195845.3
*HPRT*	for TGACACTGGGAAAACAATGCA rev GGTCCTTTTCACCAGCAAGCT	94 bp	2.05	60 °C	NM_001003357.2 NM_001313818.1

**Table 4:** Antibodies used for Western blotting.

Target	Host	Clone	Manufacturer	Dilution
ABCB1	rabbit	monoclonal EPR10364-57 (ab170904)	Abcam plc, Cambridge, UK	1/1000
RALBP1	mouse	monoclonal H-10 (sc-48337)	Santa Cruz Biotechnology, Santa Cruz, TX, USA	1/200
TOP2A	mouse	monoclonal F-12 (sc-365916)	Santa Cruz Biotechnology, Santa Cruz, TX, USA	1/200
ACTB	rabbit	monoclonal 13E5 (#4970)	Cell Signaling Technology, Leiden, Netherlands	1/1000
rabbit IgG	goat	polyclonal BA-1000	Vector Laboratories, Burlingame, CA, USA	1/1000
mouse IgG	horse	polyclonal BA-9200	Vector Laboratories, Burlingame, CA, USA	1/1000

## Data Availability

All data presented in this study are included in this article or available in the following articles: https://doi.org/10.1371/journal.pone.0230272; https://doi.org/10.1186/s12935-021-02422-9, accessed on 1 January 2023.

## Supplementary Materials

The supporting information can be downloaded at: https://www.mdpi.com/article/10.3390/ijms24098136/s1.
